# A novel statistical analysis method to improve the detection of hepatic foci of ^111^In-octreotide in SPECT/CT imaging

**DOI:** 10.1186/s40658-016-0137-4

**Published:** 2016-01-19

**Authors:** Tobias Magnander, E. Wikberg, J. Svensson, P. Gjertsson, B. Wängberg, M. Båth, Peter Bernhardt

**Affiliations:** Department of Radiation Physics, Institute of Clinical Sciences at Sahlgrenska Academy, University of Gothenburg, Gothenburg, Sweden; Department of Oncology, The Sahlgrenska Academy, University of Gothenburg, Gothenburg, Sweden; Department of Clinical Physiology, The Sahlgrenska Academy, University of Gothenburg, Gothenburg, Sweden; Department of Surgery, The Sahlgrenska Academy, University of Gothenburg, Gothenburg, Sweden; Department of Medical Physics and Biomedical Engineering, Sahlgrenska University Hospital, Gothenburg, Sweden

**Keywords:** SPECT/CT, ^111^In-octreotide, Diagnosis, Liver tumours, Neuroendocrine

## Abstract

**Background:**

Low uptake ratios, high noise, poor resolution, and low contrast all combine to make the detection of neuroendocrine liver tumours by ^111^In-octreotide single photon emission tomography (SPECT) imaging a challenge. The aim of this study was to develop a segmentation analysis method that could improve the accuracy of hepatic neuroendocrine tumour detection.

**Methods:**

Our novel segmentation was benchmarked by a retrospective analysis of patients categorized as either ^111^In-octreotide positive (^111^In-octreotide(+)) or ^111^In-octreotide negative (^111^In-octreotide(−)) for liver tumours. Following a 3-year follow-up period, involving multiple imaging modalities, we further segregated ^111^In-octreotide-negative patients into two groups: one with no confirmed liver tumours (^111^In-octreotide(−)/radtech(−)) and the other, now diagnosed with liver tumours (^111^In-octreotide(−)/radtech(+)). We retrospectively applied our segmentation analysis to see if it could have detected these previously missed tumours using ^111^In-octreotide. Our methodology subdivided the liver and determined normalized numbers of uptake foci (nNUF), at various threshold values, using a connected-component labelling algorithm. Plots of nNUF against the threshold index (ThI) were generated. ThI was defined as follows: ThI = (*c*_max_ − *c*_thr_)/*c*_max_, where *c*_max_ is the maximal threshold value for obtaining at least one, two voxel sized, uptake focus; *c*_thr_ is the voxel threshold value. The maximal divergence between the nNUF values for ^111^In-octreotide(−)/radtech(−), and ^111^In-octreotide(+) livers, was used as the optimal nNUF value for tumour detection. We also corrected for any influence of the mean activity concentration on ThI. The nNUF versus ThI method (nNUFTI) was then used to reanalyze the ^111^In-octreotide(−)/radtech(−) and ^111^In-octreotide(−)/radtech(+) groups.

**Results:**

Of a total of 53 ^111^In-octreotide(−) patients, 40 were categorized as ^111^In-octreotide(−)/radtech(−) and 13 as ^111^In-octreotide(−)/radtech(+) group. Optimal separation of the nNUF values for ^111^In-octreotide(−)/radtech(−) and ^111^In-octreotide(+) groups was defined at the nNUF value of 0.25, to the right of the bell shaped nNUFTI curve. ThIs at this nNUF value were dependent on the mean activity concentration and therefore normalized to generate nThI; a significant difference in nThI values was found between the ^111^In-octreotide(−)/radtech(−) and the ^111^In-octreotide(−)/radtech(+) groups (*P* < 0.01). As a result, four of the 13 ^111^In-octreotide(−)/radtech(+) livers were redesigned as ^111^In-octreotide(+).

**Conclusions:**

The nNUFTI method has the potential to improve the diagnosis of liver tumours using ^111^In-octreotide.

## Background

Timely and precise detection of metastatic colonies in the liver of a cancer patient is crucial if we are to make the best treatment choices [1]. Metastatic disease may indicate that a different curative regimen, or perhaps a palliative option, is now the best course of action [2–5]. Unfortunately, our ability to make these decisions is hampered by the difficulty that we face in detecting small masses, with low contrast and noise [6].

Single photon emission tomography (SPECT), using a radiolabelled mimic of somatostatin, ^111^In-octreotide, has become the established methodology with which to image somatostatin receptor (sstr)-positive tumours [7, 8] in patients with neuroendocrine tumours. Together with morphological imaging techniques, such as computed tomography (CT) and magnetic resonance imaging (MRI), it has become an important tool for tumour visualization, staging, and evaluation of somatostatin receptor status [9]. In addition, one of the major eligibility criteria for patients undergoing therapy with either ^177^Lu- or ^90^Y-labelled somatostatin analogues is that their tumour should bind more ^111^In-octreotide than normal liver [10–12]. The purpose of this study was to develop a complementary method that could help the physician in reaching their diagnosis of sstr-tumour involvement using SPECT images of the liver.

Despite significant observer variability, nuclear medicine still, to a large extent, depends on the subjective decision-making of a single physician [13, 14]. Unfortunately, quantitative analyses that are simple to use, and easy to understand for the observer, are rarely implemented in this field. Nevertheless, recent success with computer-assisted diagnosis in the detection of bone metastases [15, 16] may prompt a rethink for neuroendocrine tumours. A key aspect of these systems is their necessity for a ‘library’ of patient data; in essence, the algorithm needs to be taught how to recognize the tumour. With this input, it should be possible to develop more straightforward, and objective methodologies, with which to guide decision-making.

Due to the limited spatial resolution of the gamma camera, small tumours, with only a modest uptake of the radiolabel, will fall beneath the limit of detection. A second problem that limits resolution is noise in the system. Voxel clustering may give a false impression of a mass that has bound the radiolabel, i.e. a false positive signal is generated. The inherent loss in specificity that this generates further diminishes the chances of authentically locating a metastatic focus. Our experimental methodology is based on a statistical approach with the assumption that the distributions of uptake foci differ between healthy livers and livers with tumour involvements. Starting with the maximum voxel value within the liver, and successively calculating the number of segmented uptake foci at decreasing threshold values, a graph of the number of uptake foci, versus threshold value, is obtained. We will, in this study, demonstrate that the number of disjointed segmented uptake foci, as a function of decreasing threshold value for normal liver (with no true uptake foci), can be displayed as a symmetrical bell-shaped curve. When authentic foci are present, this curve is shifted and/or compressed towards decreased threshold values. This shift can be exploited to discriminate between authentic and artifactual foci. We then apply this method in a retrospective analysis of 53 randomly selected patients, previously diagnosed by ^111^In-octreotide SPECT to be clear of liver metastases. Within this group are a number of patients that, during a 3-year follow-up, were confirmed by other methods to display liver metastases. For these patients, we used our experimental methodology to address whether it would have helped the clinician to reach a positive ^111^In-octreotide diagnosis for liver involvement, rather than the negative diagnoses that were reported.

## Methods

### Patient cohort

**Fig. 1:**
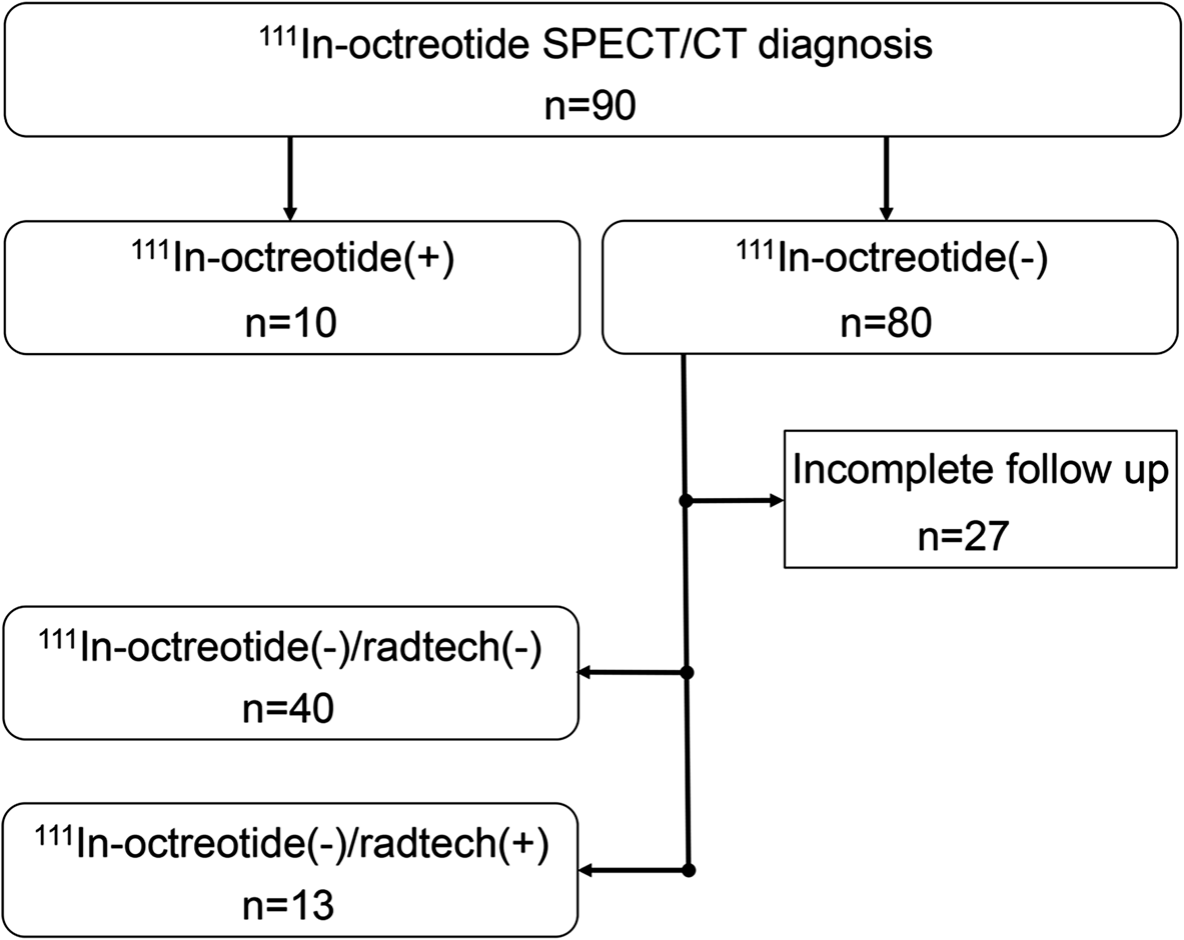
Patient flow of the ^111^In-octreotide investigations

This retrospective study was approved by the Regional Ethical Review Board in Gothenburg and performed in accordance with the Declaration of Helsinki and national regulations. For evaluation of the segmentation analysis method, 80 ^111^In-octreotide-negative (^111^In-octreotide(−)) liver patients were retrospectively selected from the examination years 2004–2011 (Fig. 1). For these years, the patient data were arranged in alphabetical order by the patient name, and a consecutive alphabetical selection was applied. In addition, 10 ^111^In-octreotide-positive (^111^In-octreotide(+)) liver patients were recruited based on tumour burden; for five, this was high; for the remaining five patients, their tumour burden was designated as minor by a qualified observer. The ^111^In-octreotide(+) livers were chosen to demonstrate the robustness of our method, and were critically important in helping us to benchmark the parameters used in our subsequent analyses. As our method is designed to guide the observer towards improved tumour detection, we retrospectively followed up the ^111^In-octreotide(−) patient cohort over 3 years, using a combination of radiological techniques (radtech), MRI, SPECT, positron emission tomography (PET)/CT, ultrasound, and CT. This screening was used to separate the ^111^In-octreotide(−) patients into two groups: one with no confirmed liver tumours (^111^In-octreotide(−)/radtech(−)) and one with confirmed liver tumours (^111^In-octreotide(−)/radtech(+)).

### Segmentation of uptake foci

Automatic segmentation of uptake foci in liver tissue at different voxel threshold values is a methodology developed for the analysis of SPECT/CT data using ^111^In-octreotide. Raw image data (128 × 128, 120 projections) were reconstructed according to the standard clinical protocol, i.e. using ordered subset expectation maximum (OSEM) reconstruction, with two iterations and ten subsets. In a departure to this protocol, the resulting volume was then unfiltered; the justification for this was to improve our chances of finding small lesions. The liver volume of interest (VOI) was segmented from SPECT, but in some SPECT investigation, the high uptake in surrounding tissues hampered the automatic segmentation. In these situations, the segmentations were performed in the CTs. The segmentations performed from the SPECT, or CT, used either an isosurface, a region growing, or a graphics processing unit (GPU) accelerated level set algorithm. Manual editing was used to refine the segmentation. The liver VOI was then thresholded at values between 0 and the maximum voxel value. At each threshold value, the number of uptake foci (NUF), i.e. the number of connected regions unlinked to other regions, was determined using a technique known as connective-component labelling [17]. Connective-component labelling is an algorithm where subsets of connected components (here, regions of connected voxels) are uniquely labelled, enabling calculation of the number of subsets, i.e. the NUF in our method.

### The normalized number of uptake foci versus threshold index (nNUFTI) method

The number of unlinked uptake foci was calculated at each threshold value, generating a distribution of NUF versus a threshold index (ThI):

1 $$ \mathrm{T}\mathrm{h}\mathrm{I}=\frac{C_{\max }-{C}_{\mathrm{thr}}}{C_{\max }} $$

where *C*_max_ is the maximal voxel value in the VOI and *C*_thr_ is the voxel threshold value. The NUF can therefore be described as a function of ThI, ranging from 0 to 1. By normalizing the NUF (nNUF) to the maximal NUF in a liver VOI, it is possible to display and then compare individual ^111^In-octreotide SPECT data (by comparing nNUF versus ThI (nNUFTI)).

### Creation of a visualization tool for the observer

A parallel aim was to integrate the nNUFTI method with a visualization tool for the observer. This was performed by incorporating a slider along the ThI-axis, which provides a real-time visual representation of the corresponding uptake foci in the 3D liver VOI. Unconnected uptake foci were assigned different colours, from a palette of 256. This enabled the observer to pinpoint suspicious lesions by closely studying their noise structure and the clustering of uptake foci. Additionally, the displayed 3D volume of disconnected uptake foci can be analysed contemporaneously with CT and MRI data. The nNUFTI programme was written in c++ and implemented into PhONSAi (the medical Physics, Oncology and Nuclear medicine image research platform at Sahlgrenska Academy). Using the PhONSAi platform, the segmentation of the liver, together with CT data processing, was performed in parallel on the graphic card (CUDA) to obtain a rapid segmentation performance. An experienced clinical observer oversaw the graphical layout of the nNUFTI programme.

### Determination of optimal value for the normalized number of uptake foci

To determine the optimal value of the nNUF that allows the greatest separation of ThI between the ^111^In-octreotide(−)/radtech(−), and ^111^In-octreotide(+) groups, the *t* score in Student’s *t* test was used. The nNUF with the highest *t* score was used to determine the ThI values in further ^111^In-octreotide analyses using the nNUFTI method.

### Analysis of the ^111^In-octreotide-negative patients with the nNUFTI method

Using the nNUFTI method, the ThI was determined for all patients using the value of nNUF as previously described. For the ^111^In-octreotide(−)/radtech(−) group, it was confirmed that ThI was dependent on the mean activity concentration; therefore, ThI was normalized (nThI) by the function that best describes this dependency. Comparison of the nThI for the ^111^In-octreotide(−)/radtech(−) and ^111^In-octreotide(−)/radtech(+) groups was conducted by Student’s *t* test. *P* < 0.05 was considered significant. Lastly, the positive predictive value (PPV) for the individual nTHI values in the ^111^In-octreotide(−)/radtech(+) group was calculated.

## Results

Of the first 80 selected ^111^In-octreotide(−) patients, 27 had to be excluded due to incomplete follow-up data. Forty of the remaining 53 patients were diagnosed as having no sign of liver tumours within their follow-up time. These were designated the ^111^In-octreotide(−)/radtech(−) group. The remaining 13 patients had their liver tumours detected by either CT or ultra sound, designated as the ^111^In-octreotide(−)/radtech(+) group.

**Fig. 2:**
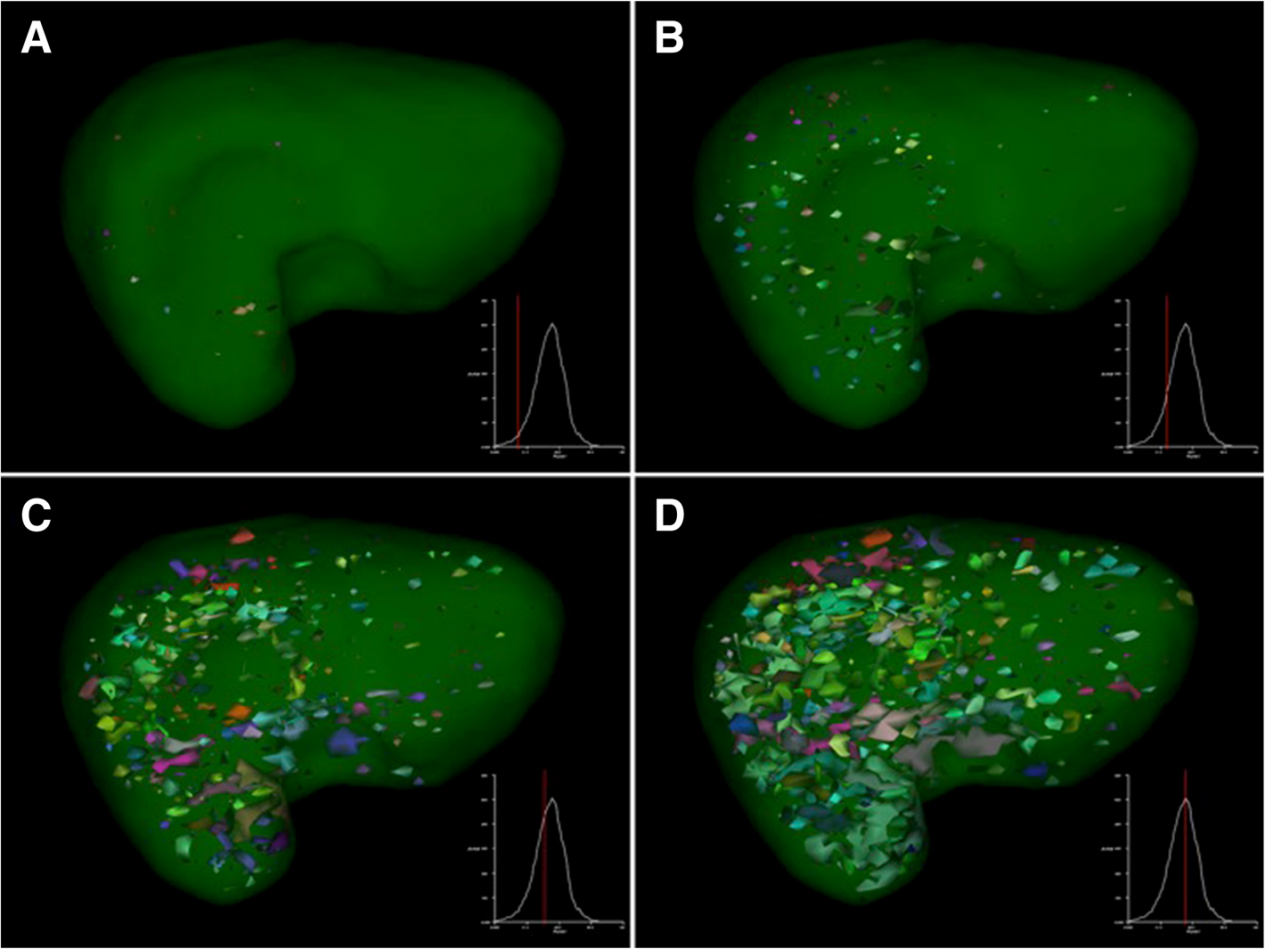
An example of the graphical layout, showing the distribution of uptake foci in a liver with no confirmed metastases (octreotide(−)/radtech(−)). The observer can freely rotate the image in all planes, allowing the precise interrogation of focal uptake. The entire liver (indicated by the *transparent green colour*) is segmented. The additional graphs, appearing at the lower right of each panel, show nNUF (*y*-axis), plotted against threshold index (ThI) (*x*-axis). Radiolabelled foci are shown coloured. In panel **a**, the ThI is set for the inspection of high intensity areas. As the ThI is increased (panels **b**, **c**, and **d**), more foci are captured, until foci begin to merge

The nNUFTI programme, for the quantification of disjointed NUF, performed well in consistently quantifying, and visualizing, NUF. Visualization analyses for our two patient groups, ^111^In-octreotide(−)/radtech(−) and ^111^In-octreotide(+), revealed a qualitative difference in the representation of their uptake foci on the graphical interface. Figure 2 shows a representative ^111^In-octreotide(−)/radtech(−) liver, with several foci simultaneously appearing on the screen. Initially, the nNUF increases continuously, in parallel with ThI. However, at a particular ThI threshold, the nNUF then decreases as the shrinkage of the inter-focus distance causes foci to merge (Fig. 2d).

**Fig. 3:**
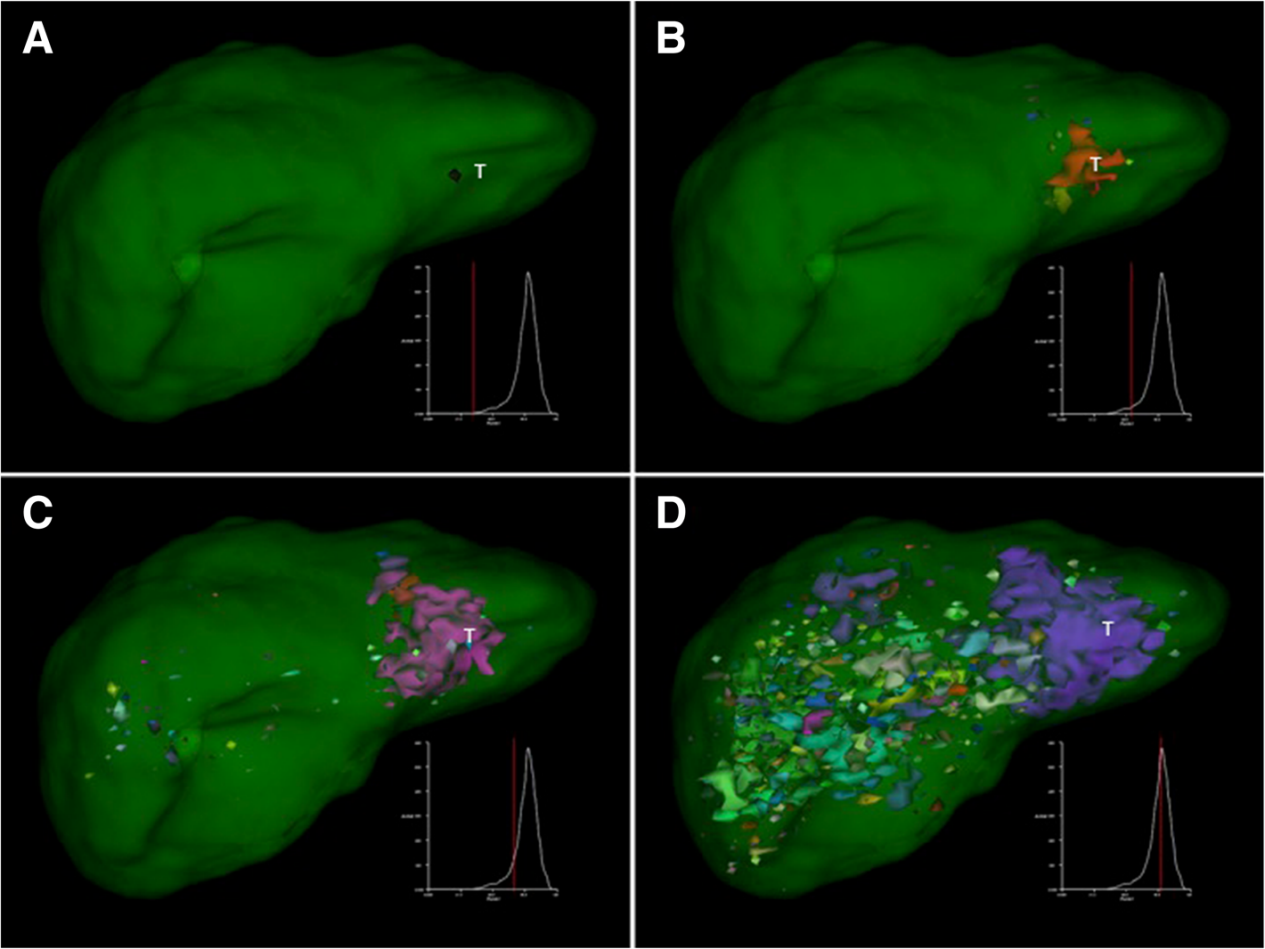
The graphical layout demonstrating uptake foci in a liver carrying a ^111^In-octreotide positive tumour (^111^In-octreotide(+)), the tumour is indicated (*T*). The additional graphs, appearing on the panels at the lower right, show the number of normalized uptake foci (*y*-axes), plotted against threshold index (ThI) (*x*-axes). In a liver with ^111^In-octreotide-positive tumours, the distribution of uptake foci will be shifted towards higher ThI, due to the merging of uptake foci in the tumour area (**a**–**d**)

In a patient confirmed to have liver tumours, and displaying a higher uptake of ^111^In-octreotide in the tumour versus normal liver tissue, the nNUF remained low, despite a prolonged increase in ThI (Figs. 2 and 3). The high radiolabel intensities in the tumour volume will result in those voxels merging into a single uptake focus, or a few uptake foci, around the tumour volume (A–B). No uptake foci aligned to ‘noise’ will appear at this level of ThI. As the maximal number of uptake foci are detected, noise correlated foci begin to appear, but to a lower degree than those in a healthy liver (Figs. 2d and 3d).

**Fig. 4:**
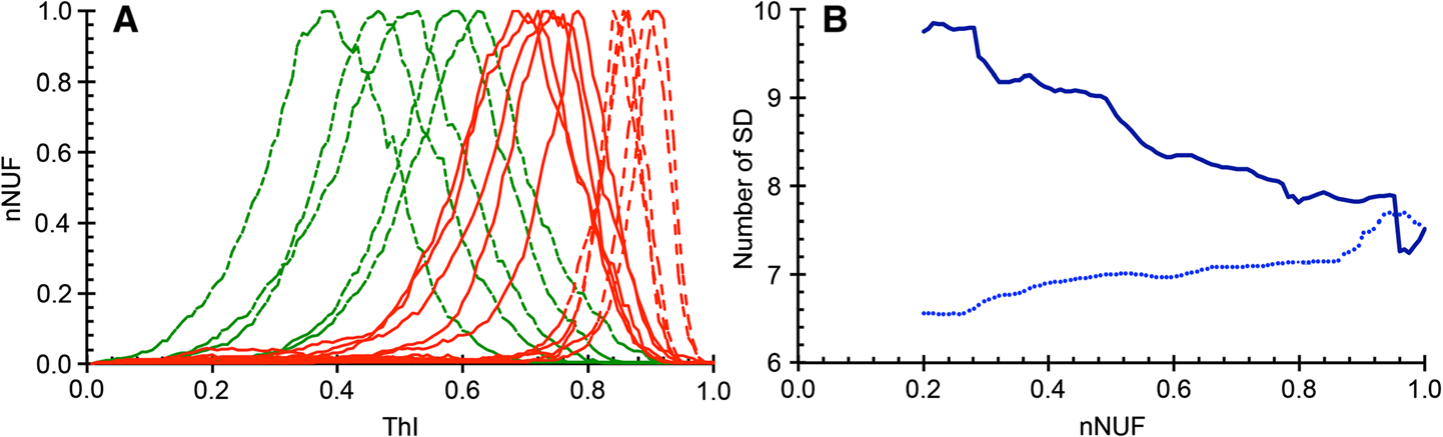
Graphical representation of the increased threshold index (ThI) delay for ten octreotide(+) livers (*red curves*). **a** The *dashed red lines* denote livers with a large tumour burden; *red lines* indicate livers with a minor tumour involvement. The *green lines* represent five octreotide(−)/radtech(−) livers. **b** The *t* scores for octreotide(+) livers (*n* = 10) and octreotide(−)/radtech(−) livers (*n* = 40) were plotted against the normalized number of uptake foci (nNUF). The two lines show the *t* scores for NUF values on either side of the maximum of the nNUFTI curve; the *lower*, *dashed*, *light blue line* shows values to the left of the curve maximum, and the *higher*, *dark blue line* shows values to the right of the peak

The nNUF versus ThI values were then plotted for five selected octreotide(−)/radtech(−) livers and ten octreotide(+) livers (Fig. 4a). For the five patients with substantial tumour involvement, there was a pronounced compression of the nNUFTI curve, in favour of increased ThI values. The remaining five, octreotide(+) livers, represent patients diagnosed with minimal detectable tumour involvement. The nNUFTI data for these patients were used to define an optimal ThI for tumour detection, i.e. the optimal separation between the ThI values for octreotide(−)/radtech(−) and octreotide(+) livers. It was demonstrated that the highest *t* scores were obtained using a threshold on the right side of the maximal nNUF value (Fig. 4b). The *t* score increased until a nNUF value of approximately 0.25. Similar results were obtained when the analysis of the octreotide(+) livers was restricted to five livers with either minor or significant tumour involvement (data not shown). The nNUF = 0.25 value, at the right side of the distribution curve, was used in further analyses of tumour detection in individual patients.

**Fig. 5:**
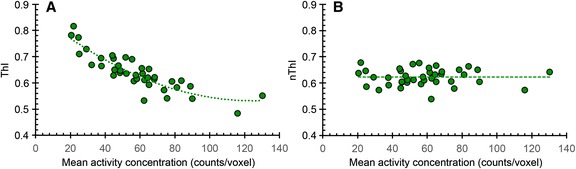
The dependence of ThI, and mean activity concentration, in octreotide(−)/radtech(−) livers. The ThI shows a strong mean activity concentration dependence (**a**). This dependence is absent when normalized to 60 counts per voxel (**b**)

For the octreotide(−)/radtech(−) livers, it was noted that ThI decreased with increasing mean activity concentration (Fig. 5a). As the parameter setting for a second-degree polynomial function best described this relationship (*r*^2^ = 0.80), we used this function to correct ThI to a mean activity concentration of 60 counts per voxel (Fig. 5b). This generated a normalized activity concentration ThI (nThI). The nThI had a mean and standard deviation of 0.623 and 0.032, respectively.

**Fig. 6:**
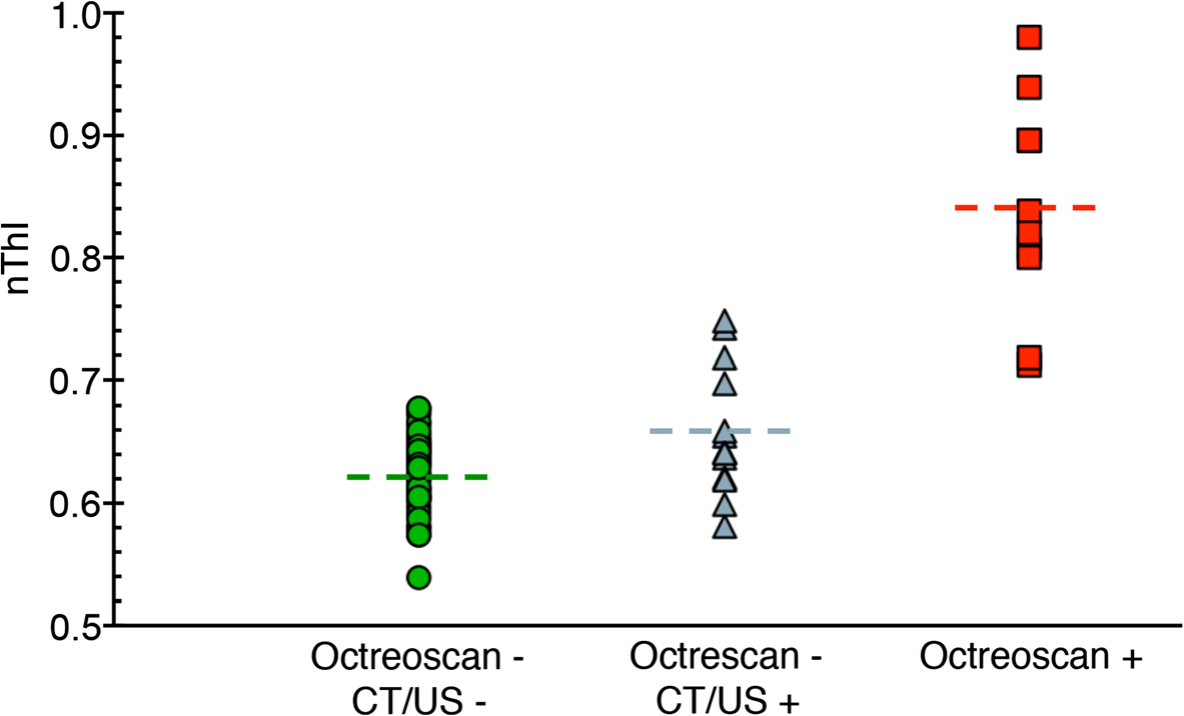
The normalized threshold index (nThI) for the octreotide(−)/radtech(−) (*green circles*), the octreotide(−)/radtech(+) group (*grey triangles*), and the octreotide(+) group (*red squares*). The *dashed vertical line* indicates the mean nThIs. The mean/standard deviation was 0.62/0.032 and 0.66/0.053 for the octreotide(−)/radtech(−) and octreotide(−)/radtech(+) group (*P* < 0.01), respectively. The highest nThI value for the octreotide(−)/radtech(−) group was 0.68. The mean/standard deviation was 0.84/0.088 for the octreotide(+) group, which clearly differs from the octreotide(−)/radtech(−) group (*P* < 0.0001)

**Fig. 7:**
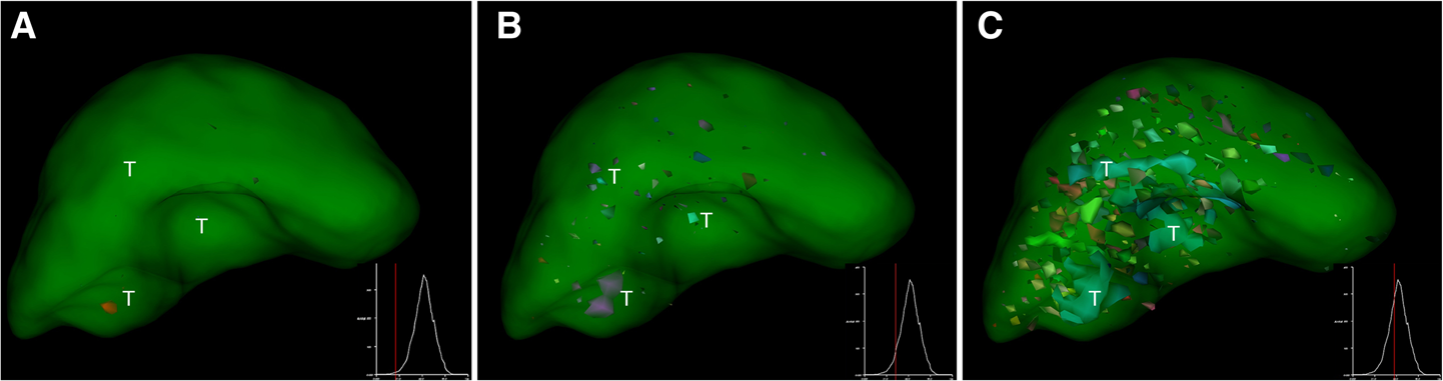
The graphical layout of the distribution of uptake foci in a liver with later confirmed tumours (*T*), i.e. an octreotide(−)/radtech(+) liver. The uptakes are not easily visualized without knowledge of the localisation site, but due to the multiple small uptake sites the distribution of uptake foci will be shifted towards higher ThI (**a**-**c**), resulting in a nNUFTI-positive liver for patient number 32F

A total of 13 patients in the 53 ^111^In-octreotide patient cohort were diagnosed with liver tumours during their 3-year follow-up (Table 1). The tumour involvement was diagnosed either with CT or ultrasound and arose from 0.8 years prior, to 2.6 years post, their negative ^111^In-octreotide diagnoses. The nThIs for this group was significantly different from those of the octreotide(−)/radtech(−) group (*P* < 0.01) (Fig. 6). Eleven of the 13 malignant livers had higher nThI values than the mean nThI value for the octreotide(−)/radtech(−) group (Fig. 6). Four of the octreotide(−)/radtech(+) livers had a higher nThI than the largest value achieved by the octreotide(−)/radtech(−) group. Consequently, the PPV for these four livers were 1.0, with a mean PPV of 0.56. Figure 7 shows the graphical layout, and the nNUF distribution, for patient 32F, which had a PPV of 1.0. All octreotide(+) livers had higher nTHI than the largest value achieved by the octreotide(−)/radtech(−) group (*P* < 0.0001).

**Table 1:** Characterization of the octreotide(−)/radtech(+) livers, including results from the nNUFTI analyses

Patient	Time of diagnosis (years)	Image modality	Tumour type	Number of metastases	nThI	PPV
42F	0.0	CT	HCC, carcinoid(ECL)	Multiple	0.748	1.0
32F	0.3	US	Midgut carcinoid	Three	0.743	1.0
41F	−0.8	CT	Midgut carcinoid	Single	0.718	1.0
55F	−0.4	CT	NET, origin unknown	Multiple	0.697	1.0
84M	1.0	CT	Midgut carcinoid	Multiple	0.659	0.50
25M	1.5	CT	Medullary thyroid cancer	Single	0.655	0.50
43 M	1.4	US, CT	Midgut carcinoid	Four	0.642	0.35
90M	2.4	CT	Medullary thyroid cancer	Single	0.641	0.38
30F	2.7	CT	Endocrine pancreatic	Single	0.637	0.38
109F	1.5	CT	Lung carcinoid	Single	0.621	0.32
111F	2.6	CT	Midgut carcinoid	Two	0.619	0.33
104F	0.2	US	Midgut carcinoid, cervix cancer	Multiple	0.599	0.27
103M	−0.1	CT	Midgut carcinoid	Multiple	0.581	0.26

## Discussion

We now report a novel and improved segmentation method, with a proven ability to produce real-time images of uptake foci, for immediate evaluation by the observer. The nNUFTI display provides the observer with a quality-control parameter for tumour involvement, with an additional probability value provided by the nThI.

The nNUFTI algorithm that we created is based on the assumption that ^111^In-octreotide-positive tumours will skew the nNUFTI curves towards higher ThI values. Our results also showed that both large ^111^In-octreotide-positive tumour burdens, as well as modest tumour involvement, could be easily detected by their compression of the nNUFTI curve. These data emphasize the value of this methodology in the early detection of metastatic liver disease. To evaluate if this methodology could outperform conventional ^111^In-octreotide imaging, we randomly selected 53 ^111^In-octreotide negative patients and provided follow-up for the detection of emergent, late, liver tumours. Thirteen of these patients were found to display tumours in CT, MRI, PET/CT, or ultra sound investigations, i.e. 25 % of the ^111^In-octreotide-negative patients were false negatives. The nNUFTI method was able to yield a statistically significant separation between the two groups, suggesting that the nNUFTI algorithm can, without bias, detect emergent liver tumours.

The main obstacles to tumour detection using SPECT imaging include difficulty in discriminating between uptake of the radiolabel by normal tissue versus the tumour due to a low tumour-to-normal activity concentration ratio (TNC), poor resolution, and noise. These confounding factors are the same, whether using conventional observation or the nNUFTI method. To mitigate these issues, we defined a quantitative measure for tumour detection by comparing 40 healthy livers with 10 ^111^In-octreotide-positive livers. Five of the tumour-positive livers were selected because they carried a modest tumour burden; these are the patients whose cancers are ordinarily most difficult to detect. We determined that a nNUF value of 0.25, at the right side of the nNUFTI curve, provided the greatest discrimination between groups. Our results also highlighted the fact that ^111^In-octreotide is unevenly distributed through the liver (Fig. 2), with the highest uptake displayed initially in the segment representing the right side of the lobe; this non-uniform distribution of activity was observed for most livers. When the TNC is constant, as compared with a regional liver activity distribution, tumours localized to a segment of high-activity concentration will compress the entire nNUFTI curve, whereas a tumour localized to a low activity concentration segment might only compress the nNUFTI curve on the right side. This non-uniformity of hepatic activity concentration increases the sensitivity of the nNUFTI method on the right side of the curve.

The influence of noise on the nNUFTI method was demonstrated by the strong nThI versus voxel signal correlation. This was corrected for, using a polynomial function. While different models can be fitted to the data, our choice was based on the best-fit approach. Future modelling studies should be performed to codify this method.

We noted a variation in the mean voxel signal for ^111^In-octreotide between livers due to different radiolabel pharmacokinetics, non-standard sensitivity of the gamma cameras, and different SPECT measurement times. In particular, we noted a marked difference for ThI when using our two gamma cameras; this introduced a 25 % difference in sensitivity for ^111^In. A depressed sensitivity increased the ThI, which then had to be corrected using the adjusted nThI. This correction eliminated the variability of the camera and the duration of measurement.

The probability estimator that we derived for tumour involvement is intended to guide the observer in their diagnostic decision-making. SPECT data with nThIs of between 0.64 and 0.69 appeared to be the most problematic in terms of obtaining an accurate diagnosis. For these patients, it is recommended that follow-up includes other tests, i.e. CT, MRI, ultra sound, PET/CT, or additional SPECT/CT. For lower nThIs, it seems most probable that additional ^111^In-octreotide investigations will be of less value, either because there are no tumours to be found or, if present, they are ^111^In-octreotide negative. A higher nThIs (>0.70) is a strong indicator of tumour burden (Fig. 6), although it may be difficult to visualize the tumours even with the developed visualization tool. Comparing Figs. 3 and 7, the tumours marked in the octreotide(−) liver in Fig. 7 are hardly detectable even with knowledge of their localization. Nevertheless, the nNUFTI method reported a value of nThI as high as 0.74, well above any radtech(−) liver and showing the sensitivity of the nNUFTI method. In contrast, the tumour in the octreotide(+) liver in Fig. 3, with an nThI of 0.84, is well visualized.

The present study followed the standard reconstruction protocols used in the clinic, i.e. OSEM with two iterations and ten subsets; ultimately, this may not be the optimal setting for the nNUFTI method. Further modelling studies using simulated tumours in the non-malignant livers, and different reconstruction settings, might be of value in determining the best reconstruction method to use with nNUFTI. Necessarily, for obtaining realistic noise, the modelling studies should be performed by Monte Carlo simulations of the tumours into the sinograms and thereafter reconstruction.

The tumours that could be detected by the nNUFTI method were also diagnosed contemporaneously with CT or ultrasound (US). Those tumours not detected by nNUFTI were only found by CT or US, 1 to 3 years after the initial ^111^In-octreotide diagnosis. These late arising tumours were not detected by a later ^111^In-octretide investigation. These data would suggest that the expression of the somatostatin receptor might be low for these tumours. Two of these tumours were medullary thyroid cancers, and one was a lung carcinoid, both known to have a lower uptake of ^111^In-octreotide [18], i.e. they were poor candidates for detection by ^111^In-octreotide.

Early detection of liver tumours is of huge benefit to the patient in terms of managing their treatment options. Current treatment for neuroendocrine tumours comprises different regimens. One used alternative, that has achieved some attention, is treatment with the radionuclide-labelled somatostatin analogues, ^177^Lu-DOTATATE and ^90^Y-DOTATOC [10, 19, 20]. One criterion for selecting patients for these treatments is that the uptake of the radionuclide is higher in the tumours than in normal liver tissue. However, this estimation is difficult to ascertain for small tumours, and therefore, these treatments tend to be biased towards patients with more advanced disease. However, it would be preferable to use these radionuclides to treat the disease at an earlier stage [21]. Future studies will also be performed to analyse whether this method can be used to follow treatment response in all NUF regions, thereby estimating the metastatic cure probabilities for liver tumours [21, 22].

While the present work focused on SPECT data of ^111^In-labelled somatostatin analogues in the liver, the method that we developed might be applicable to other volumes of interest in the patient and is also practicable for all diagnostic radionuclides, and in extension, to PET-imaging.

## Conclusions

We verified the utility of a novel method (nNUFTI) with which to convincingly detect observer defined ^111^In-octreotide-positive tumours, as well as non-visualized tumours, in SPECT images. Our data indicates that the nNUFTI algorithm has the potential to become a useful analytical tool with which to complement, and improve, the conventional diagnosis of liver tumours using ^111^In-octreotide.

## Compliance with ethical standards

This work was funded by the Swedish Cancer Society, the Swedish Radiation Safety Authority, the King Gustav V Jubilee Clinic Cancer Research Foundation, and the Swedish Federal Government under ALF agreement. This retrospective study was approved by the Regional Ethical Review Board in Gothenburg and performed in accordance with the Declaration of Helsinki and national regulations. The need for written informed consent was waived.
